# EquiSim: An Open-Source Articulatable Statistical Model of the Equine Distal Limb

**DOI:** 10.3389/fvets.2021.623318

**Published:** 2021-03-03

**Authors:** Jeroen Van Houtte, Filip Vandenberghe, Guoyan Zheng, Toon Huysmans, Jan Sijbers

**Affiliations:** ^1^imec-Vision Lab, University of Antwerp, Antwerp, Belgium; ^2^Equine Hospital Bosdreef, Moerbeke-Waas, Belgium; ^3^Center for Image-Guided Therapy and Interventions, Institute for Medical Robotics, Shanghai Jiao Tong University, Shanghai, China; ^4^Section on Applied Ergonomics and Design, Faculty of Industrial Design Engineering, Delft University of Technology, Delft, Netherlands

**Keywords:** principal component analysis, statistical shape model, morphometrics, bone shape model, equine/horse model, equine hoof, computer aided (geometric) design, digitalization 3D

## Abstract

Most digital models of the equine distal limb that are available in the community are static and/or subject specific; hence, they have limited applications in veterinary research. In this paper, we present an articulatable model of the entire equine distal limb based on statistical shape modeling. The model describes the inter-subject variability in bone geometry while maintaining proper jointspace distances to support model articulation toward different poses. Shape variation modes are explained in terms of common biometrics in order to ease model interpretation from a veterinary point of view. The model is publicly available through a graphical user interface (https://github.com/jvhoutte/equisim) in order to facilitate future digitalization in veterinary research, such as computer-aided designs, three-dimensional printing of bone implants, bone fracture risk assessment through finite element methods, and data registration and segmentation problems for clinical practices.

## 1. Introduction

Digital three-dimensional (3D) anatomical models have become an important aspect in the digitalization of veterinary research and medical practices (1). Being acquired by computed tomography (CT) imaging or magnetic resonance imaging (MRI) (2) or by 3D optical scanning (3), those subject-specific models find their way into finite element analyses (FEA) (4), augmented reality guidance during operations (5), and training of radiograph segmentation networks (6).

A standard procedure in morphological studies of equine distal limb anatomical structures focuses on one-dimensional linear or angular measures, such as hoof angle, hoof length, medial-lateral width of the phalanges, and so on, or two-dimensional measures, such as the joint surface. They are measured from radiographs (7, 8), MRI data (9), photographs (8, 9), or from *in-situ* measurements *in vivo* (10, 11) or post-mortem (12).

Another way to study morphology variations is by means of statistical shape models (SSMs), which encode the 3D shape variation of the complete bone geometry, rather than reducing the shape to a limited set of discrete measures (13). The benefit of this representation, compared to linear biometrics, is that the statistical shape variability is defined as variation modes of the geometry itself, such that it can be exploited in numerous computer vision applications. In human medical research, these (articulating) SSMs have been widely adopted for training segmentation neural networks on CT data (14, 15). The models also provide prior shape information for the reconstruction of personalized 3D models from sparse point-data (16) or from two-dimensional radiographs (17, 18), to facilitate orthopaedic computer-assisted surgeries (CAS), or to generate personalized finite element models for mechanical simulations (19, 20). Integrated in deep learning techniques, the models can discriminate between pathological cases based on morphing parameters and thereby outperforms manual subjective classification (21). The inherent geometric information can also be used to study the relationship between shape and biomechanical functions (22).

Because of additive manufacturing or 3D printing, physical models can efficiently be (re-)produced from these digital models (23). Rapid prototyping has been deployed as didactic material in anatomy classes to study anatomy besides classical dissection sessions and for training of surgery techniques as an alternative to experimental animals (24, 25). Orthopedic implant design also benefits from computer-aided design (CAD) and 3D printing, as their design can be customized (26, 27). Osteosynthesis plates can be designed specifically according to the individual anatomy prior to fabrication of the plates. CAD thereby omits inter-operative bending of the plates as is the case with off-the-shelf template designs. It has been claimed that customized implant designs, which take the shape variability into account, improve the clinical outcome (27).

Despite the many potential applications, SSMs remain underexplored in veterinary research. First, this is due to lack of availability of large collections of 3D data, from which such model can be built. Most available models are static and subject specific and are therefore less relevant for CAD. Second, there is no one-to-one relation between the variation modes of an SSM and the linear biometrics. This might complicate the interpretation of SSMs and make them less attractive for veterinarians.

In the field of equine veterinary research, we see most potential applications for SSMs to the equine distal limb. The shape of the horse's distal limb bones is an important factor in determining the horse's performance. Because the phalanges and metacarpal bones distribute the impact forces upon landing on the ground, the shape (and bone mineral density) of the bones affect how efficiently forces are distributed and subsequently determine its risk of fractures (28). It has also been observed that hoof conformation is correlated to movement asymmetry (29). Uneven foot-bearing can eventually lead to biomechanical injuries or lameness, and should be taken into account for corrective shoeing and farriery (30, 31).

The aim of this paper is to provide a workflow to generate an articulating SSM of the equine distal limb. Furthermore, the SSM's variation modes are associated with conventional linear biometrics in order to ease the model interpretation. Unlike earlier SSMs, our model describes the statistical shape variation of the different bones simultaneously in one model. This ensures correct jointspace distances for different model instances and enables articulation of the model toward different poses.

We first outline the methodology to construct an articulating multi-component statistical shape model (aSSM) of the equine distal limb, which is based on the earlier work of (17). Next, we describe the major statistical variation modes in terms of linear biometrics. In the discussion, we provide directions of future research and potential application areas in the field of veterinary research where the model can be adopted.

## 2. Materials and Methods

### 2.1. Data Collection and Data Preparation

A random collection of 70 left and right distal front limbs of 35 cold-blooded and warm-blooded horses and ponies was donated by a commercial abattoir and bulk CT-scanned post-mortem with a Canon Aquilion LB CT system (resolution: (0.78×0.78×0.5) mm^3^, tube current: 200 mA, generator power: 27 kW) from the hoof to the carpus. All legs were unshod at the time of scanning. The hoofs did not undergo prior hoof trimming or cleaning. Right limbs were later mirrored to resemble left limbs.

The acquired CT images were segmented using an open-source graph-cut multi-label segmentation technique (32), followed by minor manual corrections. The segmentation label maps were converted to digital geometry surface models by a discrete marching cube algorithm (33) and re-meshed to a coarser curvature-adaptive mesh by ACVD-software (34). Mesh artifacts were eventually resolved by MeshFix (35).

### 2.2. Construction of the Articulating Multi-Component Statistical Shape Model

**Figure 1:**
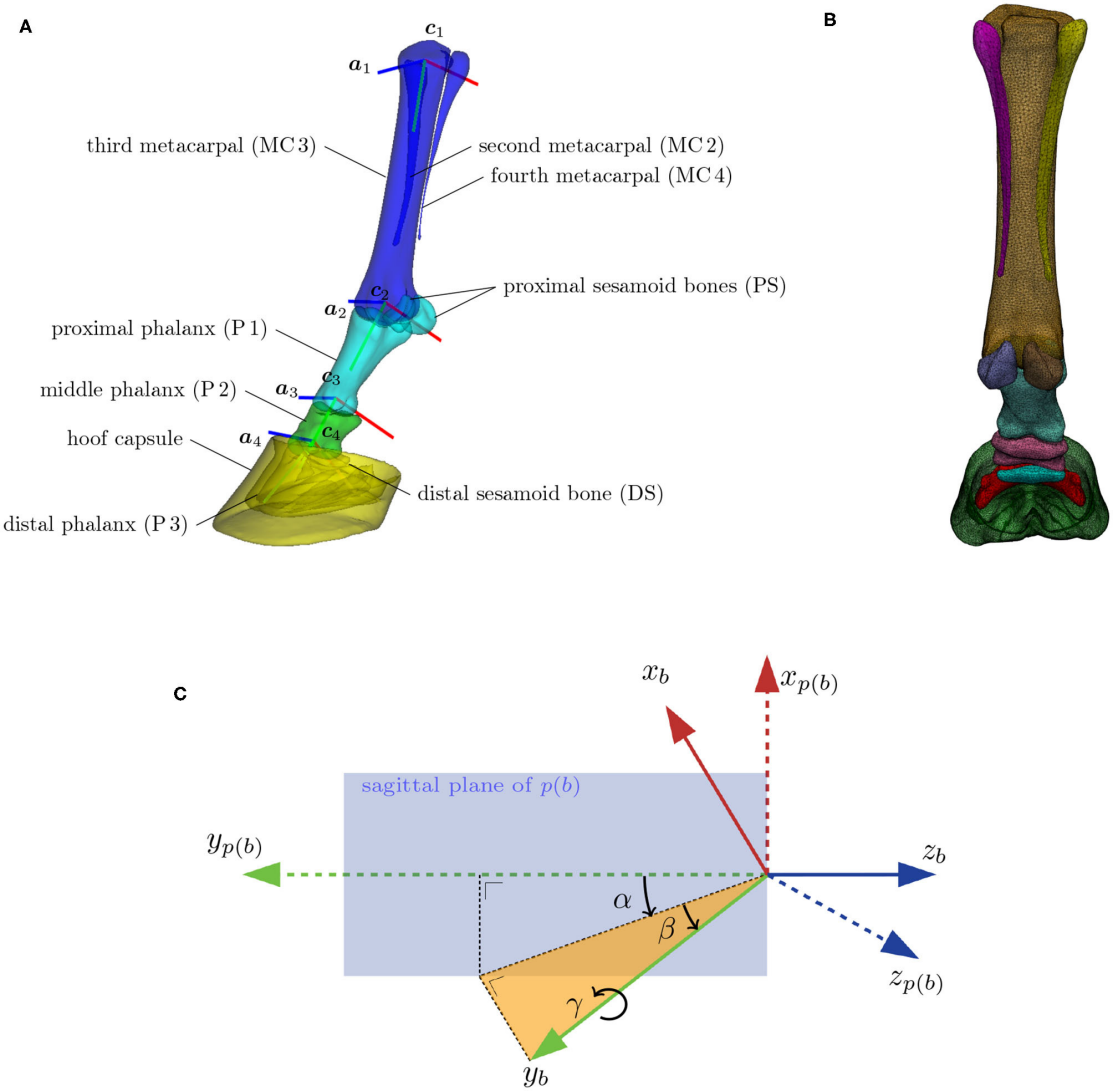
**(A)** The distal limb model consisting of nine bones and the hoof capsule. Bones with similar colors move rigidly under skeleton articulation. Each bone has a local orthogonal reference frame (***x***_*b*_, ***y***_*b*_, ***z***_*b*_) associated with it, which are here represented by, respectively, the red, green, and blue lines. The flexion/extension rotation axis of bone *b* is denoted by ***a***_*b*_ and is positioned at location ***c***_*b*_. The flexion/extension around axes ***a***_2_, ***a***_3_, and ***a***_4_ are the major degrees of freedom of the articulation model. **(B)** Model with overlayed wireframe, indicating the resolution of the surface model. **(C)** Definition of the spherical angles (α, β, γ) between a bone's reference frame (***x***_*b*_, ***y***_*b*_, ***z***_*b*_), and its adjacent parent bone's reference frame [***x***_*p*(*b*)_, ***y***_*p*(*b*)_, ***z***_*p*(*b*)_]. The extension/flexion angle α between two adjacent bones is measured inside the sagittal plane of the parent bone. The corresponding rotation axis ***a***_*b*_ coincides with the *z*-axis of the parent bone ***z***_*p*(*b*)_. The abduction/adduction angle β is measured perpendicular to the sagittal plane of the parent bone. The associated rotation axis is ***y***_*b*_ × ***z***_*p*(*b*)_. The internal rotation γ happens around the bone axis ***y***_*b*_ itself.

In this section, we describe the proposed methodology to build a compact representation model of the shape variations in our population of *L* = 70 equine distal limb models *S*_*i*_, *i* ∈ {0, …, *L*}, with *i* = 0 indicating the reference model that was adopted from earlier work (6). As illustrated in Figure 1A, each limb model *S*_*i*_ consists of *M* = 10 components (nine distal limb bones and the hoof capsule); thus, *S*_*i*_ = {*S*_*ij*_, *j* = 0, …, *M* − 1}, where *S*_*ij*_ is the *j*th component of the *i*th subject with *N*_*ij*_ vertices. We denote the homogeneous vertex coordinates of shape *S*_*ij*_ by $v_{ij}=\left\{v_{ijp}\in {\text{ℝ}}^{4},\;p=0,\ldots ,N_{ij}-1\right\}$. The number of vertices per component *j* of the reference model are tabulated in Table 1 and the resolution of the reference model is visualized in Figure 1B.

**Table 1:** Number of vertices per component of the reference model.

**Bone *j***	***N*_0*j*_**
MC 2	994
MC 3	9,159
MC 4	742
PS (lateral)	817
PS (medial)	777
P 1	4,226
P 2	2,563
P 3	2,882
DS	688
Hoof capsule	13,460

#### 2.2.1. Articulation Model

**Figure 2:**
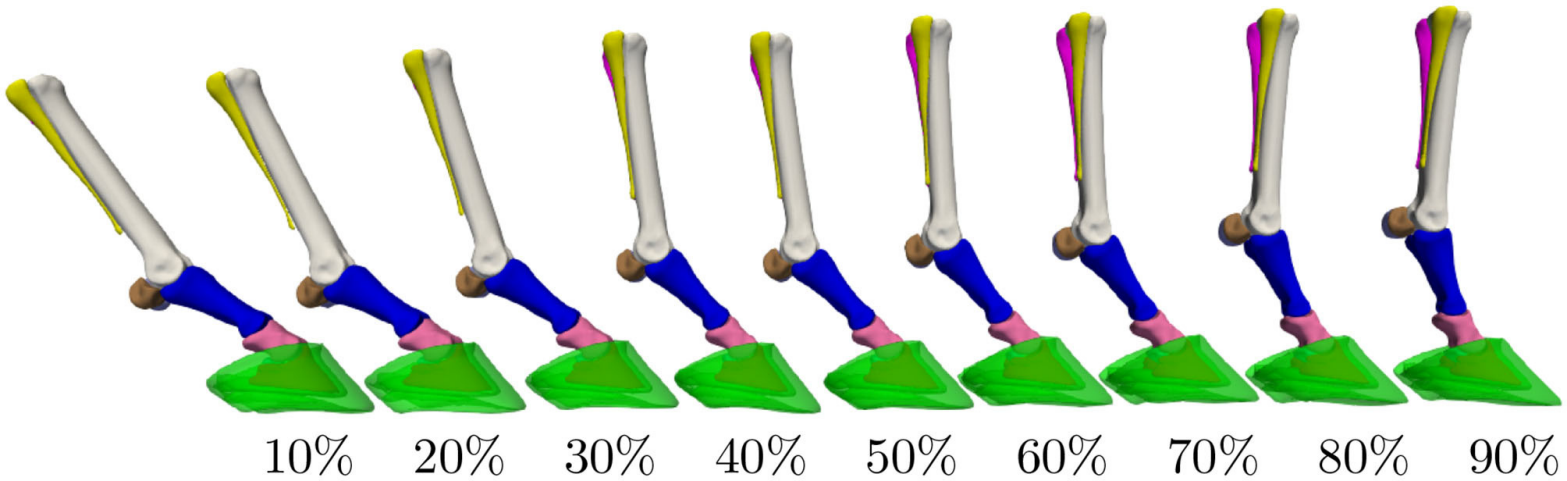
Distal limb model's articulation throughout the stance phase. The percentages indicate the completion level of the stance phase. Extension/flexion angles for the metacarpophalangeal (MCP), proximal interphalangeal (PIP), and distal interphalangeal (DIP) joints were obtained from the literature (36, 37).

Articulation of the surface model, as illustrated in Figure 2 for the different stages of the stance phase, is limited to the major degrees of freedom of the equine distal limb. This includes the extension and flexion around the following three joints: metacarpophalangeal (MCP), proximal interphalangeal (PIP), and the distal interphalangeal (DIP). The articulation model articulates the proximal sesamoid bones and the proximal phalanx as one geometry structure (38). This approximation is justified by their relatively small range of motion and any latent motion, which happens in reality, will end up as a shape variability in the model. Similarly, the distal sesamoid bone and the hoof capsule are assumed to be rigidly attached to the distal phalanx in the articulation model. Furthermore, the three metacarpal bones are rigidly attached to each other. Under these assumptions, the articulation model effectively consists of *N*_*b*_ = 4 skeleton bones to transform *M* = 10 surface model components.

The articulation model assigns a local reference frame to each of the four skeleton bones, as depicted in Figure 1A. The orthogonal reference frame is defined such that its *y*-axis aligns with the elongation axis and that its *z*-axis is perpendicular to the sagittal plane of the bone. The flexion, abduction and internal rotation angle are, respectively, identified as the three spherical coordinates (α, β, γ) between adjacent reference frames. Their definition is visualized in Figure 1C. Note that the flexion rotation axis ***a*** of bone *b* corresponds to the *z*-axis of its parent bone *p*(*b*).

To enable articulation of the distal limb bones themselves, we also define an origin ***c*** to each reference frame, which is chosen as the center of a circle, fitted to the joint surface area of the bone in its sagittal plane. The local-to-world transformation *T* ∈ ℝ^4×4^ brings the local reference frame of a bone, like the one shown in Figure 1C, to its position and orientation in world coordinates. Flexion of a bone *b* relative to its parent bone *p*(*b*) over an angle θ is obtained by the transformation $T_{p\left(b\right)}R_{z}\left(\theta \right)T_{p\left(b\right)}^{-1}$, where $R_{z}\left(\theta \right)\in {\text{ℝ}}^{4\text{×}4}$ represents a rotation over the *z*-axis. It should be noted that the articulation model is a mathematical construction and is not statistically founded by dynamic data.

#### 2.2.2. Elastic Registration

Initially, each subject *S*_*i*_ in the training database is described by its own set of vertices. To statistically describe the shape variations in this database, all shapes must be in semantic correspondence with each other, such that vertices with the same index have the same anatomical location on all training subjects. In order to do so, we elastically deform the reference component coordinates $T_{ij}T_{0j}^{-1}v_{0j}$ toward its corresponding training subject component *S*_*ij*_ and replace the training subject by the registration result, without change of notation, such that each training shape is now described by the same semantic-meaningful mesh (39).

**Figure 3:**
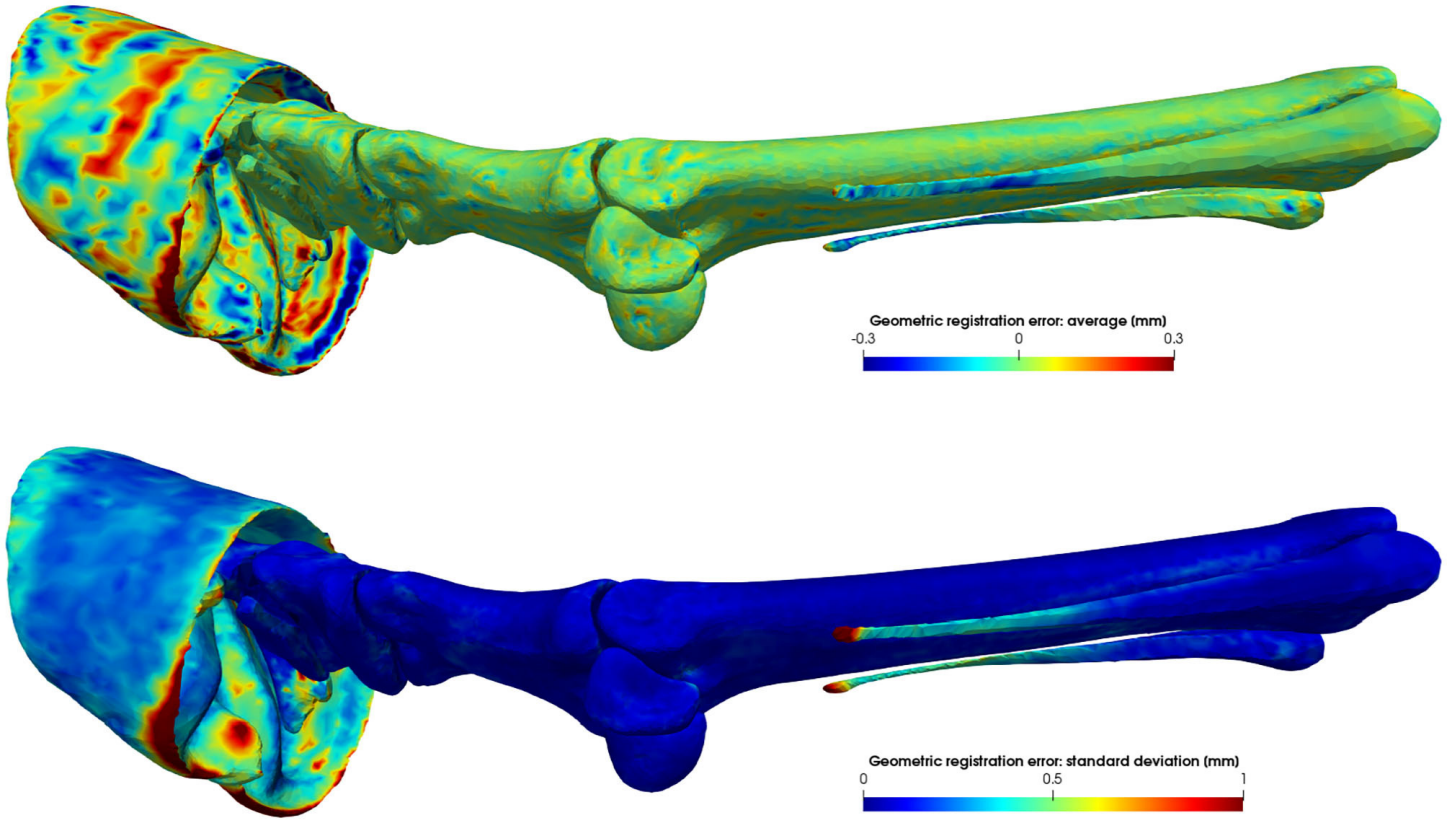
Average signed geometric error of the elastic surface registration to *L* = 70 subjects. Vertices of the registered reference model that lie inside or outside the target model have a negative or positive distance, respectively. The average error is a measure of the registration accuracy, while its variance is a measure of the precision of the registration.

In order to reduce a possible bias toward the chosen reference model, we repeat the elastic registration with the mean model as reference. Note that registered shapes are still in their original position and orientation. Replacing the subject by its registered result introduces a geometric error of how well the original surface is approximated by its registered surface. As illustrated in Figure 3, this geometric error is highly position dependent, but overall negligible. The average unsigned geometric error over the entire model equals 0.182 ± 0.002 mm.

#### 2.2.3. Scale and Pose Normalization

As we are interested in the intrinsic shape variability, we want to normalize all training subjects for their global scale. All models were scale normalized based on the length of the third metacarpal of the reference model.

Second, training subjects were originally scanned on their side in different unloaded poses, which causes unwanted pose variations in the dataset. The pose normalization of bone *b* involves finding the optimal flexion angle θ^*^, in a least-square sense, which matches the bone's geometry with the corresponding bone of the reference model in the local reference frame of its parent bone *p*(*b*):

(1) $$\begin{array}{r} \theta^{*}=\arg\underset{\theta }{\min}∥T_{0j}^{-1}v_{0j}-R_{z}\left(\theta \right)T_{ij}^{-1}v_{ij}{∥}^{2}, \end{array}$$

where the one-to-one correspondences from the previous step are exploited for the geometry matching. Note that we only optimize for the extension/flexion angle and not for abduction, adduction, and internal rotation, which are considered as remnant posture in the shape analysis.

#### 2.2.4. PCA-Based Statistical Shape Modeling

Assuming a database of *L* registered pose- and scale-normalized shapes *S*_*i*_, we can define each shape by its shape vector $s_{i}\in {\text{ℝ}}^{3F}$, which is a concatenation of its $F=\overset{M-1}{\underset{j=0}{\sum }}N_{0j}$ coordinates. The shape vectors of the *L* subjects are ordered as columns in a data-matrix *X* ∈ ℝ^3*F*×*L*^.

The goal of principal component analysis (PCA) is to find an orthogonal transformation that transforms the high-dimensional shape vectors to a low-dimensional set of linearly uncorrelated variables, which are called principal components (PCs) (13). The PCs can efficiently be calculated by first mean-centering the rows of *X* and next performing a singular value decomposition (SVD) on the low-dimensional matrix *X*^*T*^*X*. The matrix *X* multiplied by the left singular vectors of this decomposition are equal to the principal component vectors $u_{i}\in {\text{ℝ}}^{3F}$ of *X*, after normalizing the columns. The singular values of the decomposition are equal to the variances $\sigma_{i}^{2}$ of those PCs. The PCs are ordered such that the first PC accounts for the largest variation in the dataset, and each succeeding PC has the largest variance possible under the condition that it must be orthogonal to any previous component. Any shape can now be expressed as a linear combination of those PCs, weighted by its standard deviations:

(2) $$s(b)=\;\bar{s}+\sum_{i=1}^{L-1}b_{i}\sigma_{i}u_{i}$$

with $\bar{s}\in {\text{ℝ}}^{3F}$ the mean shape vector and *b*_*i*_ the contribution of the *i*th normalized PC ***u***_*i*_ to the final shape ***s***. In matrix notation, this reads:

(3) $$\begin{array}{r} s\left(b\right)=\bar{s}+EDb \end{array}$$

where the columns of matrix *E* ∈ ℝ^3*F*×*L*−1^ contain the normalized eigenvectors ***u***_*i*_ and $D=\text{diag}\left(\sigma_{1},\sigma_{2},\ldots ,\sigma_{L-1}\right)\in {\text{ℝ}}^{L-1\times L-1}$. The PC weights ***b*** ∈ ℝ^*L*−1^ allow to generate new shape instances from the SSM, different from the training data. Given a new shape ***s*** with the same topology as the SSM, the PC weights that approximate this new shape most closely are given by:

(4) $$\begin{array}{r} b=D^{-1}E^{+}\left(s-\bar{s}\right), \end{array}$$

with *E*^+^ the pseudo-inverse of the non-square matrix *E*.

#### 2.2.5. Articulating Statistical Shape Model Construction

Applying PCA to the set of registered pose- and scale-normalized shape coordinates ${ṽ}_{ij}=R_{z}\left(\theta^{*}\right)T_{ij}^{-1}v_{ij}$, it is important for each shape vector ***s*_*i*_** to also contain skeleton information besides the geometry coordinates, because both are intertwined: if the shape changes, the underlying skeleton will have to be modified as well in order to maintain proper articulation. The shape vector is therefore a concatenation of the geometry coordinates of all components of the model and the position ***c***_*b*_ and orientation ***a***_*b*_ of the rotation axes of the bones in the skeleton model, as shown in Figure 1A:

(5) $$\begin{array}{l} s_{i}=[\underset{\text{geometry coordinates}}{\underset{︸}{{\tilde{v}}_{i,0}{\tilde{v}}_{i,1}\ldots {\tilde{v}}_{i,M-1}}}\underset{\text{axis orientation}}{\underset{︸}{\log_{\mu_{1}}a_{1}^{i}\log_{\mu_{2}}a_{2}^{i}\ldots \log_{\mu_{N_{b}}}a_{N_{b}}^{i}}}\\ \;\underset{\text{axis position}}{\underset{︸}{c_{1}^{i}c_{2}^{i}\ldots \;c_{N_{b}}^{i}}}{]}^{T}\in \text{​}{\text{ℝ}}^{3F}, \end{array}$$

with $F=\overset{M-1}{\underset{j=0}{\sum }}N_{0j}+2N_{b}$. Note that we take a logarithmic map of the axis orientation vectors ***a***_*b*_ around their intrinsic means ***μ***_*b*_, because PCA assumes that the data are normally distributed in an Euclidean (high-dimensional) space. However, the rotation vectors are constrained to lie on the unit sphere *S*^2^, meaning that their length is always one and they can only vary in their orientation. Hence, the vectors lie on a curved manifold and one needs to adopt principal geodesic analysis (PGA) instead of PCA to describe the data variability (40). In short, it applies PCA on the tangent space of *S*^2^, and one needs to use the logarithmic and exponential map around the intrinsic mean to move back and forth between *S*^2^ and the Euclidean tangent space.

The reconstruction of a component *j* from the articulating SSM is obtained via:

(6) $$\begin{array}{r} s\left(b,\theta_{j}\right)=\left(T\left(b\right)◦R_{z}\left(\theta_{j}\right)\right)s\left(b\right), \end{array}$$

where ***s***(***b***) can be calculated from Equation (3). Note that the transformation *T* is also dependent on ***b*** as it depends on the rotation axis and center of the bones.

### 2.3. Biometrics

**Figure 4:**
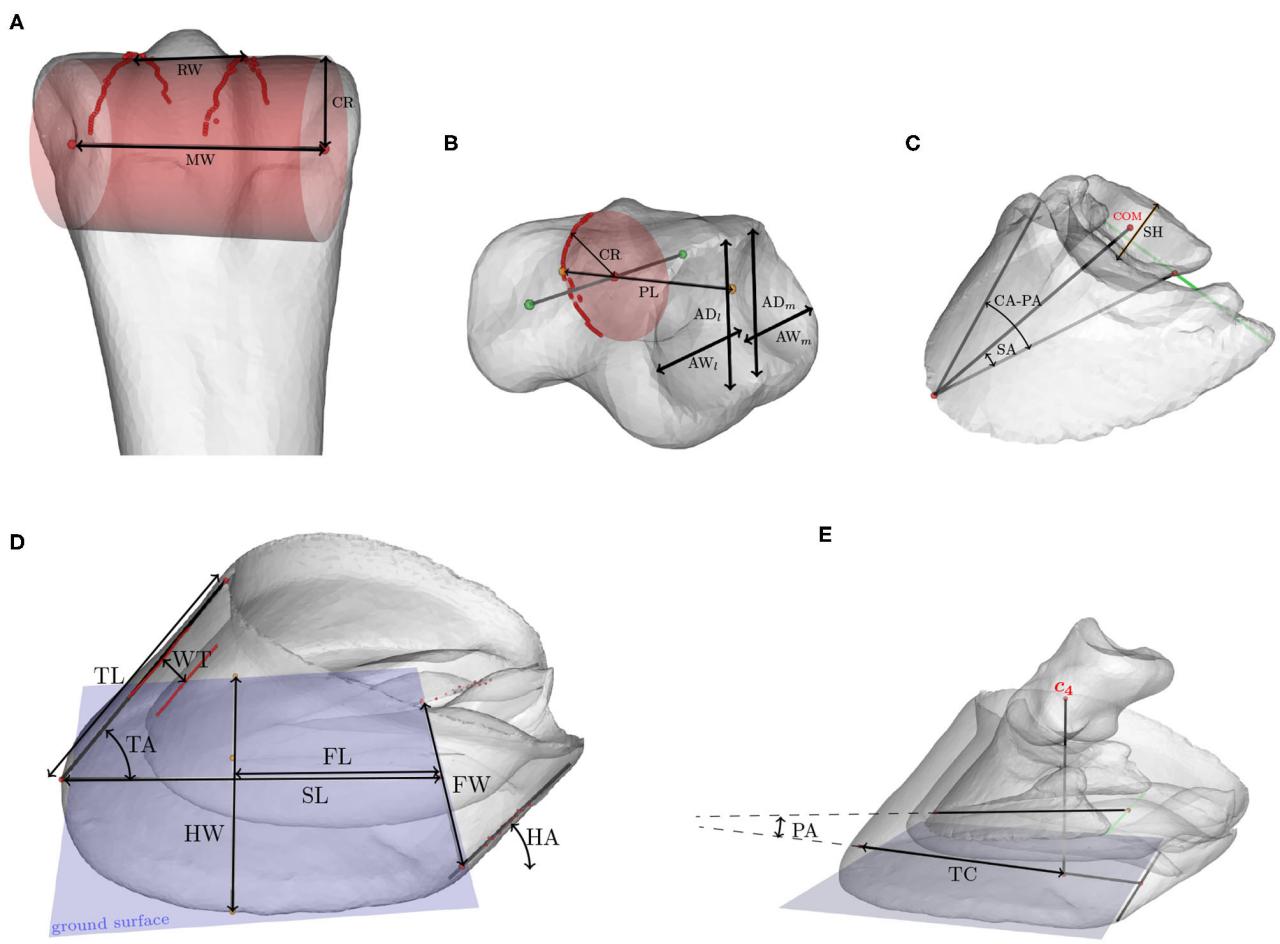
Biometrics indicated on the 3D models of **(A)** the third metacarpal, **(B)** the proximal or middle phalanx, **(C)** the distal phalanx and distal sesamoid bone, and **(D)** the hoof capsule. **(E)** shows the relative position of the distal and middle phalanx with respect to the hoof capsule. Abbreviations of the metrics are explained in Table 2.

Biometrics are linear or angular measures that characterize a component's shape and can be expressed in terms of the variation modes of the SSM. We consider biometrics for the hoof capsule, the third phalanx (7–11), the third metacarpal, the first and second phalanx (12), and the distal sesamoid bone. The definitions of the selected biometrics are tabulated in Table 2. The metrics were automatically calculated on the 3D geometry models, instead of on two-dimensional images as is often done in the literature. Figure 4 shows the biometrics indicated on the 3D models.

**Table 2:** Definition of the biometrics, indicated on the 3D models in Figure 4.

**Bone**	**Abbr**.	**Biometrics**	**Description**
Hoof capsule	FL	Frog length	Perpendicular distance between the frog apex and the palmar hoof line.
	FW	Frog width	Distance between medial and lateral heel buttress.
	HA	Heel angle	Angle between the hoof wall at the heel and the ground surface.
	HW	Hoof width	Distance between the frog apex and outer capsule wall, measured perpendicular to the sagittal plane.
	SL	Support length	Distance between toe and the palmar hoof line along the ground surface.
	TA	Toe angle	Angle between dorsal hoof wall and the ground surface.
	TL	Toe length	Distance between toe and top of capsule, along the dorsal hoof wall.
	UR	Underrun	The heel angle (HA) minus toe angle (TA).
	WT	Wall thickness	Average thickness of the hoof capsule in the cross-section of the capsule.
P 3	CA	Coffin angle	Angle between dorsal aspect of P 3 and the ground surface.
	PA	Palmar angle	Angle between palmar aspect of P 3 and the ground surface.
	CD	Capsule deviation	Coffin angle (CA) minus toe angle (TA).
	TS	Toe to heel support	Percentage of the hoof's support length (SL), which is ahead of the center of articulation ***c*_4_** of P 3, i.e.,: TS=TC/SL.
P 1, P 2	CR	Joint curvature radius	Radius of a circle fitted to distal joint surface (lateral view).
	AD	Articular surface depth	Depth of the proximal articular surface, averaged between medial (AD_*m*_) and lateral side (AD_*l*_).
	AW	Articular surface width	Width of the proximal articular surface, averaged between medial (AW_*m*_) and lateral side (AW_*l*_).
	PL	Phalanx length	Length, along major axis in sagittal plane
MC 3	CR	Joint curvature radius	Radius of a circle fitted to distal joint surface (lateral view).
	MW	Medio-lateral width	Width of the distal joint, measured along flexion rotation axis.
	RW	Sagittal ridge width	Average width of the sagittal ridge.
DS	SA	Distal sesamoid angle	Angle between the palmar aspect of P 3 and the line connecting the tip of P 3 with the distal sesamoid's center of mass.
	SH	Distal sesamoid height	Distance between distal and proximal border of the distal sesamoid bone in the sagittal plane.

#### 2.3.1. Correlation Between Biometrics and PC Modes

In order to change the shape (i.e., changing the PC weights) as a function of the biometric, we applied a multivariate linear regression between the set of PC weights ***b*** and the biometric value *k*:

(7) $$\begin{array}{r} b\left(k\right)=\left[\begin{array}{cc} \alpha & \beta \end{array}\right]\left[\begin{array}{c} 1\\ k \end{array}\right]. \end{array}$$

The regression coefficients ***α***, ***β*** ∈ ℝ^*L*−1^ represent the offset and slope, respectively. They are determined from simulated model instances (*N* = 1, 000), created from the SSM by using Equation (6) following its multivariate normal distribution.

**Figure 5:**
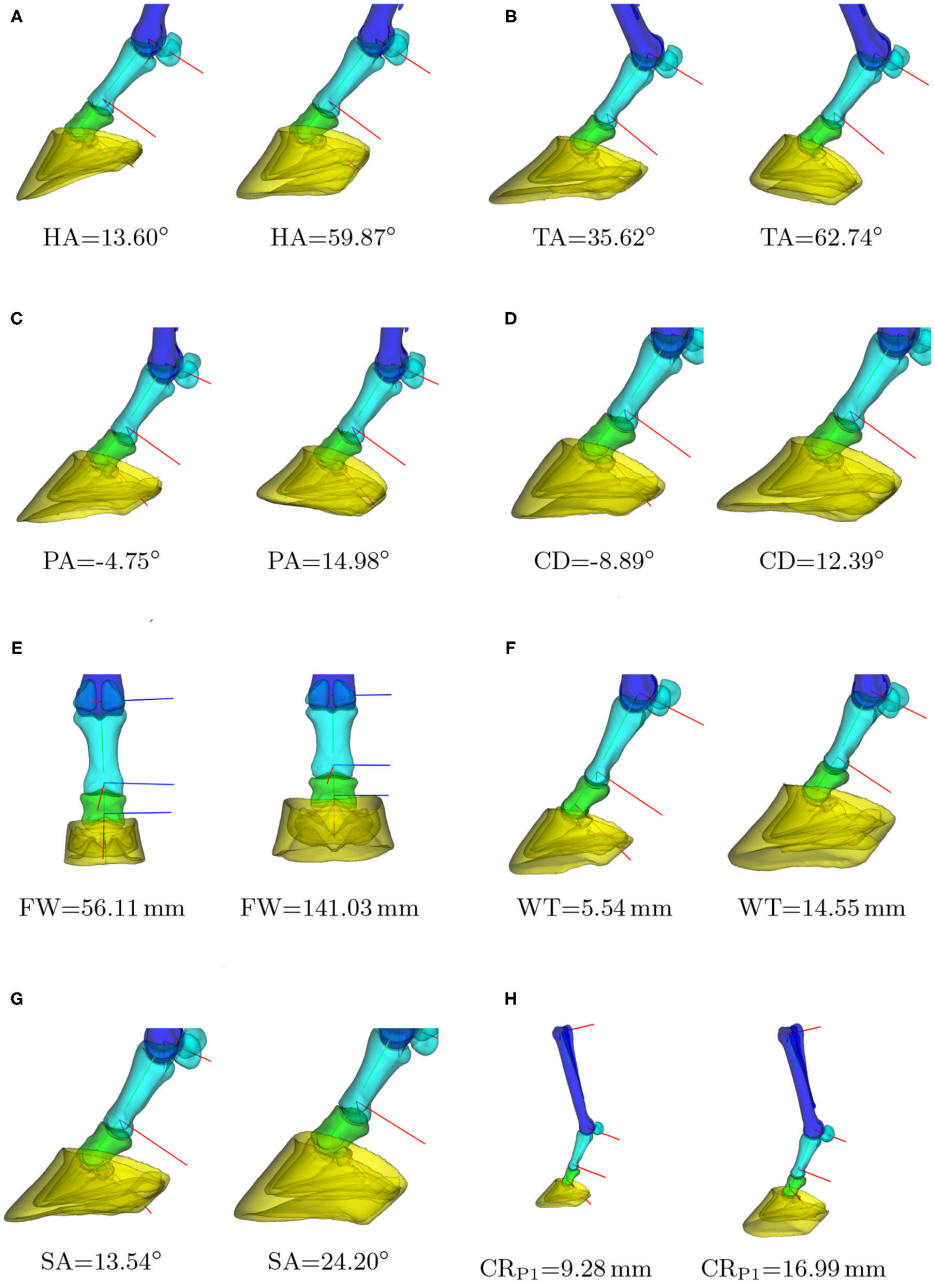
Instances of the statistical shape model (SSM) for different biometric values. For each biometric *k*, the models are shown that correspond to *k* = μ − 3σ and *k* = μ + 3σ, with μ and σ the average and standard deviation of the biometric in our dataset. Clipped models are shown to draw the reader's attention to the hoof area. In case of the curvature radius **(H)**, one can notice the global scaling of the phalanges and hoof capsule with respect to the third metacarpal. **(A)** Heel angle, **(B)** toe angle, **(C)** palmar angle, **(D)** capsule deviation, **(E)** frog width, **(F)** hoof wall thickness, **(G)** distal sesamoid angle, **(H)** P1: joint curvature radius.

Given a biometric value, the linear regression estimates the PC weights, from which the corresponding SSM's instance can be reconstructed by Equation (6). Figure 5 shows a number of model instances corresponding to the biometric values μ − 3σ and μ + 3σ. Obviously, changing one biometric also changes other biometrics as they are all correlated with each other. In order to visualize the correlations between the different biometrics, we show the Pearson's correlation coefficients in Table 3.

**Table 3:**
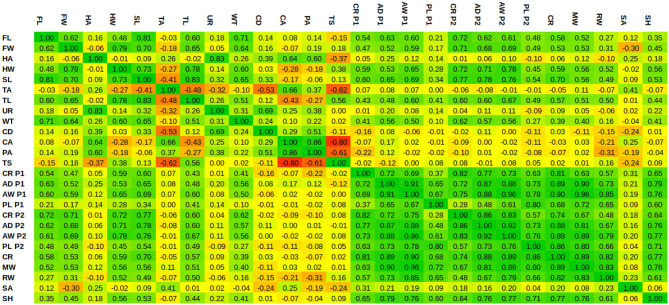
Autocorrelation table of the biometrics.

*Correlation values of −1 (red) mean that the corresponding biometrics are anti-correlated, 0 (yellow) means no correlation, and +1 (green) means positive correlation. Abbreviations of the metrics are explained in Table 2*.

## 3. Results

### 3.1. Model Performance

**Figure 6:**
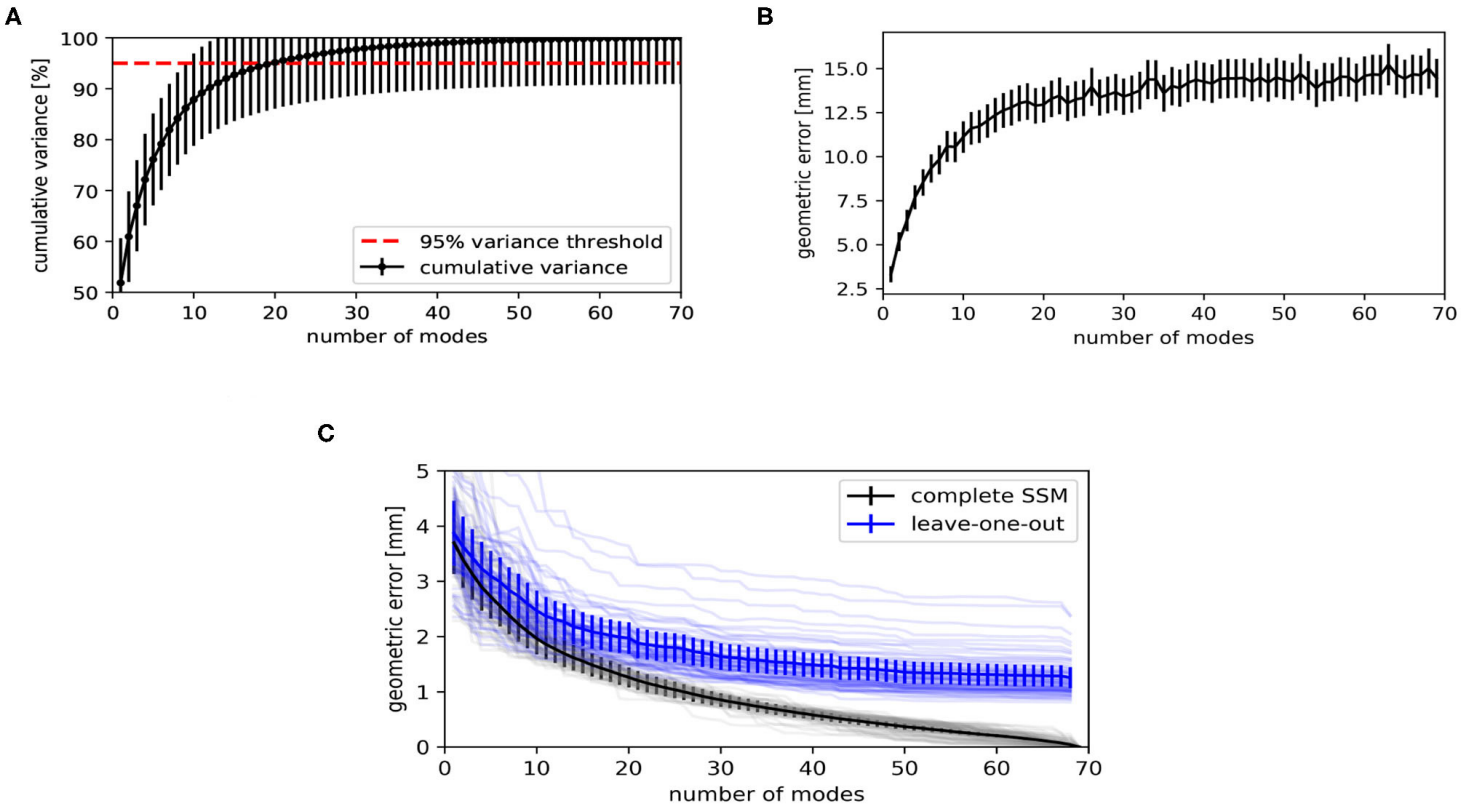
Evaluation metrics of the statistical shape model, as a function of number of principal component (PC) modes. **(A)** The compactness shows the cumulative variance explained by the statistical shape model (SSM). **(B)** The specificity indicates how similar randomly generated instances are to the training shapes. **(C)** Geometric error when fitting the SSM to unseen shapes (generalizability, blue) and to training shapes on which the SSM was built (reconstruction error, black). The faint lines show the geometric error per subject. The solid lines show the average of all subjects.

The statistical shape model's performance was evaluated in terms of compactness, generalizability, and specificity (41, 42) and are shown in Figure 6 as a function of number of PC modes. The compactness shows the cumulative variance explained by the statistical modes. Figure 6A indicates that the first 20 modes describe 95% of the variability in the training dataset. The model's specificity, shown in Figure 6B, is the extent to which the model can generate instances of the object that are close to those of the training set. To calculate this measure, random samples of the SSM have been generated by using Equation (6), according to its multivariate normal distribution. Each model instance has been compared to its closest training shape in the shape space, in terms of the root mean square error of the distance between corresponding vertices.

The generalizability indicates how well the model can be generalized to new subjects. A leave-one-out test has been performed for calculating this measure. The SSM has been rebuilt for *L* − 1 subjects and Equation (4) has been used to fit the obtained model to the subject being left out. Figure 6C shows the root mean square error between the subject being left out and the fitted result. We also show the reconstruction error when fitting to the same subject with the complete model, i.e., when the subject to which has been fitted is included in the training data. The discrepancy between both curves is the effect of fitting to unknown subjects. When using the model to fit to new instances, one might expect an average geometric error of 2.0 ± 0.3 mm when using the first 20 PC modes, as can be concluded from Figure 6C.

### 3.2. Biometrics

The generation of model instances based on a biometric value, as illustrated in Figure 5, exploits the multivariate regression relation given in section 2.3.1. The linear assumption of this regression has been evaluated by recalculating the biometric value $\tilde{k}$ on the model instance ***s***(***b***(*k*), θ_*j*_), created with the biometric value *k*. The absolute difference between the value *k* used to generate the model and the recomputed value $\tilde{k}$ on this model results in a confidence interval on *k*, which is reported in Table 4 and gives a measure for the nonlinearity of the relation between PC weights and the biometric.

**Table 4:** Confidence intervals for the biometrics.

**Biometric**	**Min**	**Max**
FL [mm]	−1.26	1.41
FW [mm]	−4.46	2.84
HA [°]	−3.34	0.780
HW [mm]	−0.415	−0.224
SL [mm]	−1.27	1.18
TA [°]	−0.502	2.00
TL [mm]	−0.897	0.196
UR [°]	−5.25	1.02
WT [mm]	−0.298	0.156
CD [°]	−1.74	0.882
CA [°]	−0.150	1.37
PA [°]	−0.628	1.04
TS [%]	−0.0231	0.00745
CR_P1_ [mm]	−0.0812	0.0225
AD_P1_ [mm]	−0.0925	0.213
AW_P1_ [mm]	−0.0593	0.0726
PL_P1_ [mm]	−0.0201	0.00
CR_P2_ [mm]	−0.00163	0.0131
AD_P2_ [mm]	−0.0929	−0.0189
AW_P2_ [mm]	−0.170	0.151
PL_P2_ [mm]	−0.0220	−0.0153
CR [mm]	−0.000740	0.0430
MW [mm]	−0.150	0.119
RW [mm]	−0.166	0.116
SA [°]	−0.455	0.443
SH [mm]	−0.0457	0.0211

Nonlinear effects have dominantly been observed for the angle biometrics, especially for the heel angle and the under-run. Despite the nonlinearity, the accuracy of the other angular biometrics is less than two degrees for the full range of [μ − 3σ, μ + 3σ]. Most linear biometrics are sub-millimeter accurate. Only the frog width, frog length, capsule deviation, and the support length of the hoof capsule have a larger difference between the expected and measured biometric.

## 4. Discussion

**Figure 7:**
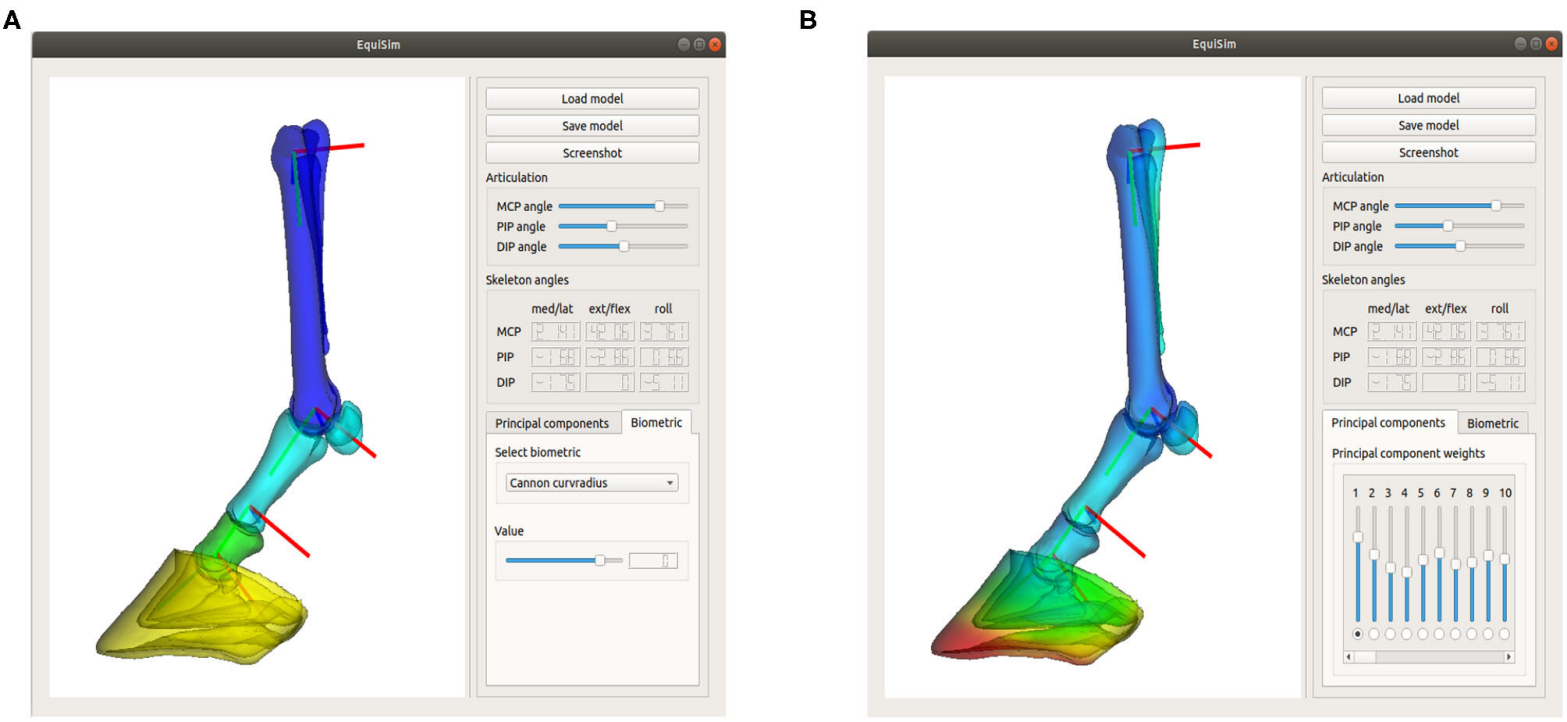
Screenshot of the graphical user interface “Equisim,” which allows the user to interact easily with the model, by either changing the biometric values **(A)** or by directly changing the PC weights **(B)**.

In this paper, a workflow has been presented to build an articulating SSM of the left equine distal limb based on principal component analysis in a pose-normalized coordinate system. As a proof of concept, the workflow has been illustrated on a dataset of 70 cadaver limbs. The resulting model describes morphological variations, while it can be articulated toward any possible pose. We found that 20 modes were sufficient to describe 95% of the population's variability and that it can be registered to new, unseen limbs with a registration accuracy of 2.0 ± 0.3 mm. To ease the morphological interpretation of the resulting statistical modes and to facilitate future research, we have explained the modes in terms of common biometrics and made the model publicly available through a graphical user interface (GUI)^1^, as shown in Figure 7. The source code of the GUI is developed in C++ on Linux.

Although the resulting model has been shown to be a compact representation of the population, there are still some limitations that can be a starting point for future research. First, the dataset of equine limbs collected for this proof-of-concept study was ill-controlled, combining different breeds, ages, levels of hoof conditions, etc. This maximized the sample size, but at the same time, made it less relevant for morphological studies. Depending on the target application, it would be beneficial to build a model from a specific dataset.

Second, the authors recommend basic hoof trimming and cleaning prior to the data acquisition. The poor hoof conditions in our collection of limbs caused ambiguities in the data segmentation and most likely also affected the segmentation accuracy, besides the reported registration accuracy. This subsequently can lead to irrelevant variation modes in the SSM. Similarly, bone ossification between MC 3 and MC 2 or between MC 3 and MC 4 (splints) also posed difficulties in the segmentation of both metacarpals in some cases.

Besides the data preparation, the model has some more theoretical limitations. The model is not a statistical shape and pose model, in the sense that the pose variations are not statistically described. In this paper, the pose of the model is altered based on a mathematical model, which does not correlate with the shape instance. Changing PC weights does not affect the range of motion. This limitation is due to the difficulty to acquire geometry data in different poses, preferably *in vivo*.

Furthermore, the model is a surface model and not a volumetric model. In order to apply our model for finite element analyses (43), one still needs to extend the model to a voxelized or tetrahedral model and assign material properties to its cells. The shape variability of our model would enable easy repetitions of the FE analysis for different shape instances. Changing only one biometric allows to study the effect of it on a particular FE result. For kinematical studies, the model can possibly be extended to a musculoskeletal model by transferring muscle, tendon, and ligament attachments from the 3D Horse Anatomy of Biosphera software (3, 44).

The main purpose of the model, as presented in this paper, lies in the compact description of the bones statistical variability. This geometric information can be exploited in CAD of different types of orthopaedic implants, suiting different classes of bone shapes. The ability to create different instances of the SSM also enables the generation of extensive training databases for deep learning applications. Digital or after 3D printing, the model can potentially have educational purposes as well.

## 5. Conclusion

In this paper, we presented a workflow to build an articulating statistical shape model of the equine distal limb, as a way to describe its morphological variations in a compact representation. 3D shape variations have been related to common one-dimensional biometrics. We thereby bridged the gap between current morphology studies and future digitalizations in veterinary research. Being available through an open-source application, our model can be an added value in veterinary anatomy classes and can potentially support future research in CADs, finite element analyses, and deep learning-based solutions for image processing tasks.

## Data Availability Statement

The statistical model and GUI presented in this study can be found on the github repository: https://github.com/jvhoutte/equisim.

## Ethics Statement

Ethical review and approval was not required for the animal study because the collection of equine limbs was donated by a commercial abattoir. The animals were slaughtered for reasons unrelated to this study. Because of the nature of the samples, no additional ethical approval was required for this study. Written informed consent for participation was not obtained from the owners because the livestock animals were slaughtered at the commercial abattoir and samples were donated post-mortem. The samples were anonymized before they got delivered to the researchers.

## Author Contributions

All authors were involved in the study design. FV was responsible for the experimental part of the study and the data-collection of the CT-data. The theoretical part of the study design was outlined by JS, TH, GZ, and JV. The data analysis and the development of the GUI was performed by JV.

## Conflict of Interest

The authors declare that the research was conducted in the absence of any commercial or financial relationships that could be construed as a potential conflict of interest.

## Footnotes

^1^https://github.com/jvhoutte/equisim
